# Effects of Cu Doping on the Microstructure, Room-Temperature Desulfurization Performance and Reaction Mechanism of Nano-ZnO

**DOI:** 10.3390/molecules31081362

**Published:** 2026-04-21

**Authors:** Yue Gao, Chunhong Shao, Xuan Qi, Junfeng Zhang, Xingqian Liu

**Affiliations:** 1 College of Chemical and Materials Engineering, Hainan Vocational University of Science and Technology, Haikou 571126, China; 2 School of Chemistry and Chemical Engineering, Hainan University, Haikou 570228, China

**Keywords:** nano-CuO/ZnO, desulfurization, room-temperature, mechanism

## Abstract

A nano-CuO/ZnO desulfurizer was successfully prepared via a homogeneous precipitation method, and the effects of Cu doping on its microstructure, oxygen species, desulfurization performance, and reaction mechanism were systematically investigated. The results show that an appropriate Cu doping amount (TZ2, Cu:Zn = 1:18.40) significantly reduces the particle size (to ~10.9 nm) compared with pure ZnO (14.3 nm), leading to an increased number of surface-active sites. XPS and TG analyses reveal that Cu incorporation increases the proportion of lattice oxygen and decreases the concentration of oxygen vacancies, indicating that the modification effect of Cu dominates over the particle size effect in regulating surface oxygen species. Despite the reduced oxygen vacancy concentration, the desulfurization performance is markedly enhanced, with TZ2 exhibiting the longest breakthrough time under oxygen-free conditions at room temperature. This improvement is attributed to the strong interaction between highly dispersed Cu species and the ZnO matrix, which promotes H_2_S adsorption and activation. Mechanistic studies demonstrate that, unlike pure nano-ZnO, where oxygen vacancy-mediated reactions dominate, the CuO/ZnO system follows a chemisorption-driven pathway involving the formation of copper sulfides and highly reactive polysulfide intermediates. Furthermore, the presence of oxygen significantly influences the reaction behavior, with an optimal oxygen concentration (~10%) maximizing desulfurization performance by balancing the generation of reactive oxygen species and sulfur intermediates. This work provides new insights into the design of high-performance ZnO-based desulfurizers and highlights the critical role of Cu-induced mechanism transformation.

## 1. Introduction

Hydrogen sulfide (H_2_S) is a highly toxic and corrosive gas widely encountered in industrial processes such as natural gas purification, petroleum refining, and coal gasification [1,2,3]. Its emission not only poses serious environmental and health hazards but also leads to equipment corrosion and catalyst deactivation, making efficient removal of H_2_S critically important for sustainable industrial development. Traditional desulfurization technologies are predominantly operated at high temperatures; however, these processes suffer from high energy consumption, severe corrosion, and secondary pollution. Consequently, the development of efficient low- or room-temperature desulfurization technologies has attracted increasing attention.

Among various desulfurization materials, zinc oxide (ZnO) has been widely recognized as an effective sorbent due to its high sulfur affinity, chemical stability, and relatively low cost [4,5,6,7,8,9]. In particular, nano-sized ZnO exhibits superior desulfurization performance compared to bulk materials owing to its large specific surface area, abundant surface defects, and enhanced adsorption capacity [10,11,12]. Previous studies have demonstrated that the desulfurization performance of nano-ZnO is strongly dependent on particle size and surface structure. Smaller particle sizes provide more active sites and oxygen vacancies, which can significantly enhance H_2_S adsorption and facilitate redox reactions leading to the formation of sulfur species such as elemental sulfur and polysulfides [3,13]. Most existing studies primarily focus on the effects of particle size and oxygen vacancies. However, nano-ZnO with small particle size and high oxygen vacancy concentration often suffers from poor stability in practical applications, which limits the development of effective strategies for further enhancing its room-temperature desulfurization performance [3,13].

In addition to ZnO-based materials, various other desulfurization sorbents have been widely investigated, including activated carbon, metal oxides (e.g., Fe_2_O_3_, MnO_x_), and composite materials. Activated carbon exhibits high surface area and adsorption capacity but often suffers from limited regeneration ability [3]. Transition metal oxides provide strong redox activity but may require elevated temperatures for optimal performance [4]. Compared with these materials, ZnO-based sorbents offer a good balance between sulfur affinity, stability, and cost, making them promising candidates for room-temperature desulfurization [3,4].

In recent years, modification of ZnO with transition metals such as Cu, Fe, Co, Ni, and Mn has emerged as an effective strategy to enhance its catalytic and adsorption properties. These transition metals can regulate the electronic structure, surface chemistry, and defect characteristics of ZnO, thereby improving its reactivity toward sulfur-containing species [13]. Copper oxide (CuO), as a transition metal oxide with excellent redox properties and strong affinity toward sulfur species, has been widely used as an active component or promoter in catalytic systems. It has been reported that Cu-based sorbents exhibit high desulfurization activity, especially under medium-to-high-temperature conditions [9,14]. However, their application in room-temperature desulfurization remains relatively underexplored. Introducing Cu into ZnO may not only modify the electronic structure and surface properties of ZnO but also provide additional active sites for H_2_S adsorption and reaction.

Cu doping can significantly influence the physicochemical properties of ZnO, including particle size, surface-active sites, and oxygen vacancy concentration, as reported in previous studies on metal–ZnO systems [10,15]. On the one hand, the dispersion of amorphous copper oxide on the ZnO surface can inhibit grain growth and reduce particle size, thereby increasing the number of surface-active sites and enhancing adsorption capacity. On the other hand, Cu species can directly participate in redox reactions with H_2_S, forming various copper sulfides and facilitating the formation of polysulfide intermediates [16]. Moreover, the synergistic interaction between Cu and ZnO may lead to a distinct desulfurization pathway compared with pure ZnO, particularly under oxygen-free and room-temperature conditions.

Various synthesis methods have been reported for CuO/ZnO desulfurizers, including co-precipitation, impregnation, and sol–gel methods. Among these, the homogeneous precipitation method enables better control over particle size and dispersion of active components, which is critical for enhancing catalytic performance. In particular, achieving highly dispersed Cu species on the ZnO surface is essential for maximizing the synergistic interaction between Cu and ZnO [17,18].

Despite these advances, several key issues remain unclear, particularly regarding the balance between surface-active sites and oxygen vacancies, as well as their respective roles in determining desulfurization performance.

Recent studies have also highlighted the importance of metal–support interactions in Cu-based desulfurization systems. For example, a recent report demonstrated that tuning the interaction between Cu species and oxide supports can significantly enhance sulfur removal efficiency and alter the reaction pathway. This finding is consistent with the mechanism transformation observed in the present study [18].

Therefore, in this study, a nano-CuO/ZnO desulfurizer was prepared via a homogeneous precipitation method, and the effects of Cu doping on its microstructure, oxygen species, and room-temperature desulfurization performance were systematically investigated. Particular attention was paid to clarifying the relationship between particle size, surface-active sites, and oxygen vacancy concentration. Although previous studies have suggested that smaller particle sizes generally lead to an increase in oxygen vacancies, our results demonstrate that Cu doping increases the proportion of lattice oxygen and suppresses the formation of oxygen vacancies, indicating that the electronic and structural modification induced by Cu species dominates over the particle size effect. Furthermore, the desulfurization mechanism under oxygen-free conditions was explored in detail. It was found that, despite the reduced oxygen vacancy concentration, the desulfurization performance is significantly enhanced due to the strong interaction between Cu species and the ZnO matrix. This interaction promotes the adsorption and activation of H_2_S and leads to the formation of highly reactive polysulfide intermediates. As a result, the reaction pathway shifts from the conventional oxygen vacancy-mediated mechanism in pure nano-ZnO to a chemisorption-driven, Cu-assisted mechanism. This work provides new insights into the role of Cu doping in regulating both surface structure and reaction pathways, offering guidance for the design of efficient room-temperature desulfurization materials.

## 2. Results and Discussion

### 2.1. Effect of Cu Doping on the Structure of the Desulfurizer

The atomic ratios of Zn and Cu in the nano-CuO/ZnO desulfurizers were determined by atomic absorption spectroscopy, as summarized in Table 1. The samples were denoted as TZ1, TZ2, TZ3, and TZ4 according to different molar ratios of CuO to ZnO. As shown in Table 1, the Cu:Zn molar ratios in TZ1, TZ2, TZ3, and TZ4 were 1:50, 1:18.40, 1:11.06, and 1:6.58, respectively.

**Figure 1:**
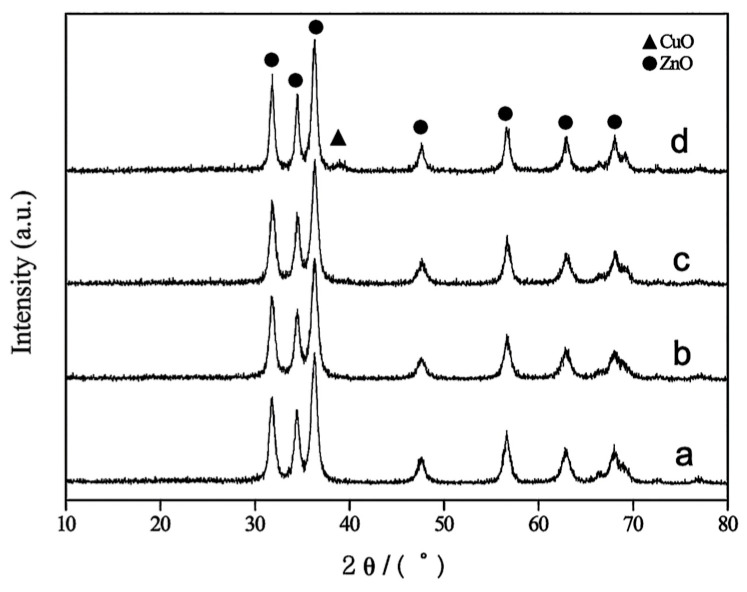
The XRD patterns of nano-CuO/ZnO desulfurizers with different Cu:Zn molar ratios (TZ1–TZ4). (a) 1:50; (b) 1:18.40; (c) 1:11.06; (d) 1:6.58.

XRD patterns of samples TZ1, TZ2, TZ3, and TZ4 are presented in Figure 1. As shown, Cu doping does not significantly alter the diffraction peak profiles of ZnO, and no new crystalline phases are observed in samples TZ1, TZ2, and TZ3. Only the characteristic diffraction peaks of ZnO are detected, indicating that Cu species are not present as an independent crystalline phase on the ZnO surface [16,19].

However, when the Cu:Zn atomic ratio increases to 1:6.58 (sample TZ4), a distinct diffraction peak appears at 2θ = 38.8°, corresponding to the CuO (111) crystal plane. Another characteristic peak of CuO may overlap with the ZnO (101) diffraction peak, making it difficult to distinguish.

At low Cu doping levels (TZ1–TZ3), no characteristic diffraction peaks of CuO are observed in the XRD patterns, indicating that Cu species are highly dispersed within the ZnO matrix, likely in an amorphous or sub-nanometric state. With increasing Cu loading (TZ4), the appearance of CuO diffraction peaks confirms the formation of crystalline CuO phases. In contrast, at higher Cu loadings, Cu tends to segregate and crystallize as CuO, forming a separate crystalline phase.

The average crystallite sizes of samples TZ1, TZ2, TZ3, and TZ4 were calculated to be 11.2, 10.9, 11.4, and 16.2 nm, respectively. For comparison, nano-ZnO prepared at the same calcination temperature (260 °C) exhibited an average particle size of 14.3 nm. The average crystallite size of the samples was calculated using the Scherrer equation based on the ZnO (002) diffraction peak. The peak broadening observed after Cu doping indicates reduced crystallite size and increased structural disorder. These results indicate that Cu doping significantly influences the particle size of the desulfurizer.

When dopant species are dispersed in an amorphous or highly dispersed state on the surface of ZnO crystallites, they can act as physical and energetic barriers that inhibit atomic diffusion and suppress particle aggregation and grain growth, as widely reported in metal oxide systems [10]. For samples TZ1, TZ2, and TZ3, CuO exists predominantly in an amorphous and highly dispersed state on the ZnO surface, resulting in smaller particle sizes compared to pure ZnO. In TZ1, the low Cu content leads to a limited inhibition effect on grain growth, and thus only a slight reduction in particle size is observed. In contrast, although TZ3 contains a higher amount of amorphous CuO, possible aggregation or non-uniform dispersion may weaken its inhibitory effect, leading to a slight increase in particle size. Notably, in TZ2, amorphous CuO is uniformly dispersed on the ZnO surface, resulting in significant peak broadening in XRD patterns and the most effective suppression of grain growth, yielding the smallest particle size.

However, for sample TZ4, Cu species are present as a separate crystalline CuO phase rather than being highly dispersed. As a result, the inhibitory effect on ZnO grain growth diminishes, and the particle size increases significantly. These findings demonstrate that both the dispersion state (amorphous vs. crystalline) and the distribution uniformity of CuO play crucial roles in determining the particle size of nano-CuO/ZnO desulfurizers.

To provide direct evidence for the variation in surface-active sites, the specific surface area and pore structure of the samples were analyzed by BET measurements, and the results are summarized in Table 2.

As shown in Table 2, the Cu-doped samples (TZ1, TZ2, TZ3, and TZ4) exhibit significantly higher specific surface area and pore volume than pure nano-ZnO (46.86 m^2^·g^−1^ and 0.3447 cm^3^·g^−1^, respectively). In particular, the TZ2 sample shows the highest specific surface area (102.34 m^2^·g^−1^) and pore volume (0.4735 cm^3^·g^−1^), indicating that Cu incorporation effectively increases the number of accessible surface-active sites. Meanwhile, the average pore diameter decreases with increasing Cu content, suggesting a refinement of the pore structure.

**Figure 2:**
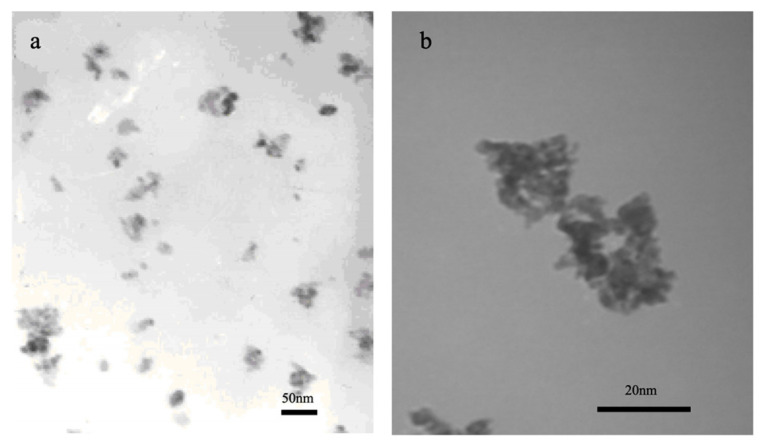
TEM images of (**a**) nano-CuO/ZnO (TZ2) and (**b**) pure ZnO (14.3 nm).

Furthermore, the morphology and particle size of the samples were characterized by TEM (Figure 2). As observed in the TEM images, the nano-CuO/ZnO (TZ2) particles are well dispersed with no obvious aggregation, and the average particle size is approximately 10–12 nm. In contrast, the particle size of pure nano-ZnO prepared under the same conditions is about 14.3 nm.

These results are consistent with the XRD analysis, which shows that Cu doping suppresses grain growth. The reduced particle size, together with the increased specific surface area and pore volume, confirms that Cu incorporation leads to a higher density of surface-active sites, thereby providing direct experimental support for the enhanced desulfurization performance.

XPS analysis of nano-CuO/ZnO (TZ2) and pure ZnO (14.3 nm) is summarized in Table 3. The Zn 2p binding energy of TZ2 is 1021.74 eV, which is shifted by 0.04 eV toward lower binding energy compared to that of pure ZnO (1021.78 eV), indicating that Zn predominantly exists in the Zn^2+^ oxidation state in the CuO/ZnO system. Furthermore, the proportion of lattice oxygen in TZ2 reaches 74.88%, which is 16.82% higher than that of pure ZnO (58.06%). The increased lattice oxygen content reduces the Zn-to-lattice oxygen atomic ratio to 1.008, approaching the stoichiometric value, suggesting a decrease in oxygen vacancy concentration after Cu doping.

This observation is consistent with the thermogravimetric (TG) analysis results obtained under an O_2_ atmosphere, further confirming that Cu incorporation effectively modifies the surface oxygen structure and reduces oxygen vacancies in the nano-CuO/ZnO desulfurizer.

**Figure 3:**
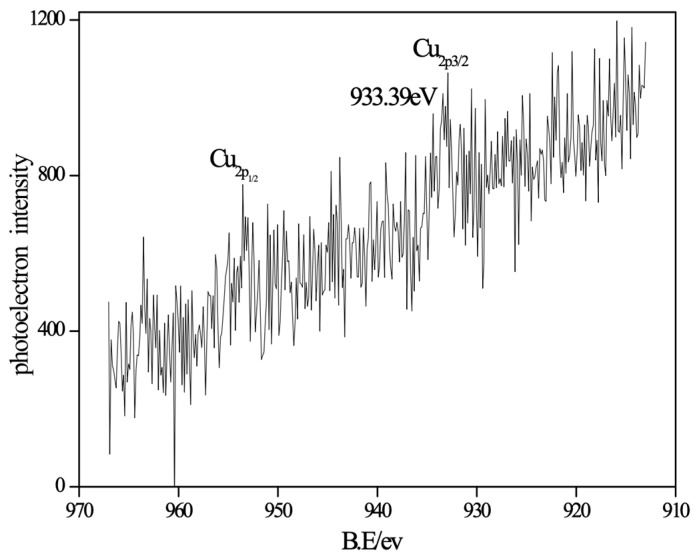
XPS spectra of Cu 2p for nano-CuO/ZnO (TZ2).

To minimize the interference from oxygen signals, XPS analysis of Cu species on the surface of the Cu-doped sample TZ2 was conducted using a Mg Kα radiation source (Figure 3). As shown in Figure 3, the Cu 2p signal is relatively weak, and the Cu 2p_3_/_2_ binding energy is located at 933.39 eV. Notably, the characteristic satellite peaks typically associated with Cu^2+^ species are barely observable.

Combined with the XRD and atomic absorption results, this phenomenon can be attributed to the low Cu loading in sample TZ2, which leads to a limited amount of CuO on the surface of the desulfurizer and consequently weak XPS signal intensity. Therefore, the absence of distinct Cu^2+^ satellite features does not necessarily indicate the absence of Cu^2+^ species. Instead, it is more likely due to the low concentration and high dispersion of Cu species on the ZnO surface.

Based on the Cu 2p_3_/_2_ binding energy, it can be inferred that Cu is still present in an oxidized state, although its spectral features are not prominent.

**Figure 4:**
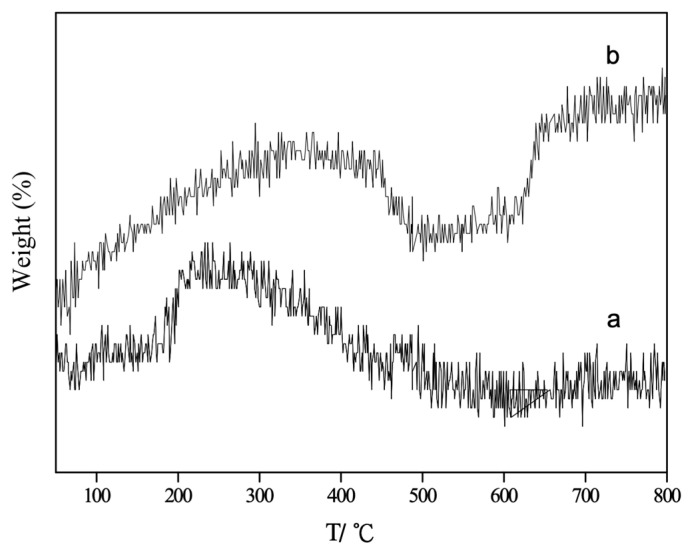
TG curves of nano-ZnO and nano-CuO/ZnO (TZ2) under an O_2_ atmosphere. (a) nano-ZnO; (b) nano-CuO/ZnO.

Thermogravimetric (TG) analysis of nano-ZnO and nano-CuO/ZnO (TZ2) desulfurizers was conducted under an O_2_ atmosphere to further investigate the effect of Cu incorporation on the structural properties and oxygen species of the materials. By comparing the oxygen adsorption behaviors of ZnO and nano-CuO/ZnO (TZ2), the relationship between oxygen vacancies in the lattice and adsorbed oxygen species was indirectly evaluated (Figure 4). The measurements were performed under an O_2_ atmosphere with a heating rate of 10 K·min^−1^ from room temperature to 1073 K.

As shown in Figure 4, pure ZnO exhibits a broad weight loss over the temperature range of 236–590 °C, whereas the nano-CuO/ZnO (TZ2) sample shows weight loss within a narrower and higher temperature range of 370–560 °C. This shift toward higher temperatures after Cu doping suggests a change in the nature and stability of oxygen species on the surface. Since the TG measurements were carried out under an oxygen atmosphere, the observed weight loss is primarily associated with the desorption or transformation of oxygen species within the desulfurizer.

The interaction between gaseous oxygen and metal oxide surfaces generally proceeds through a series of transformations, including molecular adsorption and stepwise reduction to reactive oxygen species (e.g., O_2_ (g) →O_2_ (ads) →O_2_^−^ →O_2_^2−^ → O^−^ →O^2−^), with the stability of these species increasing progressively along this sequence [20]. The relative concentration of these intermediate species depends strongly on oxygen partial pressure and temperature, and their desorption temperatures increase accordingly.

Based on previous studies on ZnO-based and metal oxide systems, the weight loss observed in the range of 236–320 °C for ZnO can be attributed to the desorption of chemisorbed oxygen species, which are typically released at relatively low temperatures (~300 °C). In contrast, the weight loss in the range of 370–560 °C is associated with the removal of more stable quasi-lattice or lattice oxygen species.

For nano-ZnO, the presence of abundant oxygen vacancies facilitates oxygen adsorption under an O_2_ atmosphere, leading to the formation of a significant amount of chemisorbed oxygen species [20]. However, after Cu doping, XPS results (Table 3) indicate an increase in lattice oxygen proportion and a decrease in oxygen vacancy concentration. As a result, the amount of chemisorbed oxygen species is reduced, and the TG behavior of TZ2 is dominated by the desorption of more stable quasi-lattice oxygen species rather than weakly bound chemisorbed oxygen.

Therefore, the combined TG and XPS results consistently demonstrate that Cu incorporation significantly modifies the surface oxygen structure and reduces oxygen vacancy concentration in the nano-CuO/ZnO desulfurizer [16].

### 2.2. Effect of Cu Doping on Desulfurization Performance

#### 2.2.1. Effect of Cu Loading on the Desulfurization Performance of Nano-CuO/ZnO

**Figure 5:**
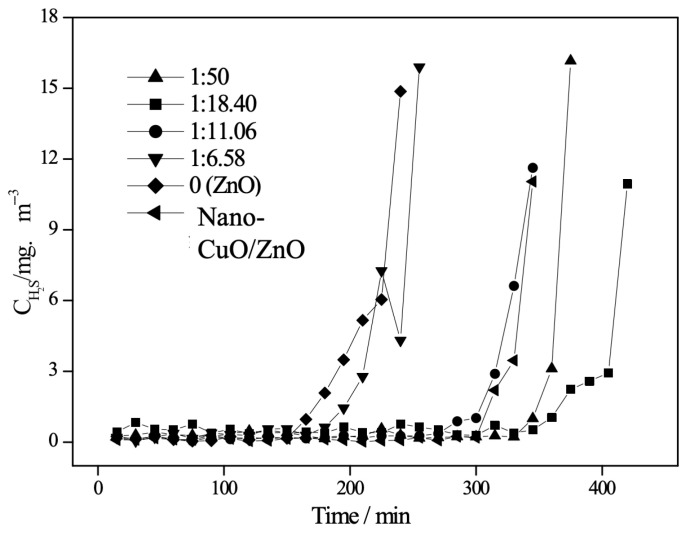
The effect of Cu on the desulfurization performance of nano-CuO/ZnO with different Cu:Zn atomic ratios (1:50, 1:18.40, 1:11.06, and 1:6.58), along with pure ZnO and mechanically mixed samples.

The influence of CuO loading on the desulfurization performance of nano-CuO/ZnO was evaluated at room temperature and atmospheric pressure, with a gas hourly space velocity (GHSV) of 3000 h^−1^, an inlet H_2_S concentration of 1852 ± 0.1 mg·m^−3^, and in the absence of oxygen. The results are presented in Figure 5. The mechanically mixed sample was prepared by physically grinding pre-synthesized nano-CuO (7 nm) and nano-ZnO (14.3 nm) powders (obtained from our previous studies) in an agate mortar to achieve the desired Cu:Zn atomic ratio [17].

As shown in Figure 5, the desulfurization activity follows the order: TZ2 > TZ1 > TZ3 > TZ4, and all Cu-doped samples exhibit superior performance compared to pure nano-ZnO, indicating that the incorporation of CuO significantly enhances the desulfurization activity.

According to the XRD results (Section 2.1), Cu incorporation leads to a reduction in particle size, which increases the specific surface area and the number of surface-active sites. It has been widely reported that smaller particle sizes provide more exposed active sites and enhance adsorption and reaction processes, thereby improving desulfurization performance [20].

At low Cu loadings (TZ1 and TZ2), CuO is highly dispersed on the ZnO surface, which effectively inhibits grain growth and promotes the exposure of active sites. Meanwhile, the strong interaction between highly dispersed Cu species and the ZnO matrix contributes to improved catalytic performance, which is consistent with previous studies on Cu–ZnO systems [17].

However, further increasing the Cu loading leads to the segregation of CuO as a crystalline phase (as observed in TZ4), which diminishes its dispersion and weakens the interfacial interaction between CuO and ZnO. As a result, the synergistic effect between Cu species and ZnO is reduced, leading to decreased desulfurization activity. Similar behavior has been reported in Cu-based desulfurization materials, where excessive loading results in particle aggregation and reduced active site accessibility [14].

Therefore, an optimal Cu loading exists, where the balance between high dispersion, strong metal–support interaction, and sufficient active sites leads to the best desulfurization performance.

To further clarify the role of Cu–ZnO interaction, a comparison was made between nano-CuO/ZnO (TZ2) and a mechanically mixed sample consisting of 7 nm CuO and 14.3 nm ZnO with the same Cu:Zn atomic ratio (1:18.40). The nano-CuO/ZnO prepared via the precipitation method exhibits significantly higher desulfurization performance than the mechanically mixed counterpart. This result demonstrates that the enhanced activity of TZ2 is not a simple additive effect of CuO and ZnO, but rather arises from the strong interfacial interaction and synergistic effect between Cu species and the ZnO matrix, which facilitates H_2_S adsorption and subsequent reaction.

#### 2.2.2. Effect of Oxygen Partial Pressure on the Desulfurization Performance of Nano-CuO/ZnO (TZ2)

**Figure 6:**
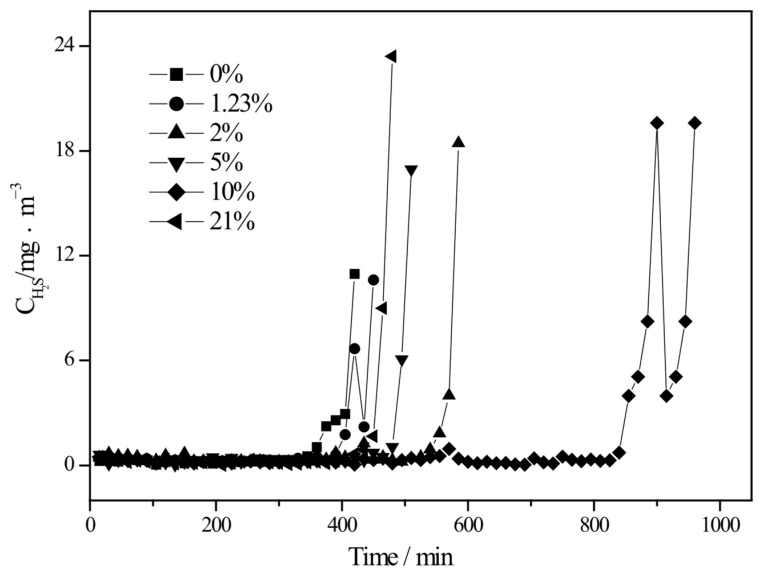
Effect of oxygen partial pressure on the desulfurization performance of nano-CuO/ZnO (TZ2).

The effect of oxygen partial pressure on the desulfurization performance of nano-CuO/ZnO (TZ2) was investigated at room temperature and atmospheric pressure, with a gas hourly space velocity (GHSV) of 3000 h^−1^ and an inlet H_2_S concentration of 1852 ± 0.1 mg·m^−3^. The results are shown in Figure 6.

As illustrated in Figure 6, when the oxygen content in the reaction gas increases from 0% to 1.23% and 2%, the desulfurization activity (breakthrough time) increases accordingly. This indicates that the presence of oxygen promotes the desulfurization process. However, with a further increase in oxygen concentration, the activity does not continue to improve monotonically. The desulfurization performance follows the order: 21% < 5% < 2% < 10%, with the highest activity observed at an oxygen concentration of 10%.

This behavior suggests that oxygen plays a dual role in the desulfurization process. At low oxygen concentrations, the amount of reactive oxygen species generated on the catalyst surface is insufficient to sustain efficient oxidation of H_2_S, thereby limiting the desulfurization performance. As the oxygen content increases to an optimal level (around 10%), the generation of active oxygen species (such as O_2_^−^ and O^−^) is enhanced, facilitating the oxidation of H_2_S to elemental sulfur and polysulfides, and thus significantly improving desulfurization efficiency.

However, excessive oxygen concentrations lead to a decline in performance. This can be attributed to the inhibition of effective interactions among reactants, products, and the catalyst surface. High oxygen partial pressure may suppress the formation and transformation of reactive sulfur intermediates by reducing the collision probability between H_2_S, intermediate sulfur species, and active sites. Additionally, excessive oxygen may alter the surface reaction pathway, leading to the over-oxidation of intermediates or blocking of active sites.

Therefore, an optimal oxygen partial pressure exists in the nano-CuO/ZnO system, where a balance is achieved between the generation of active oxygen species and the effective formation of reactive intermediates, resulting in maximum desulfurization performance.

**Figure 7:**
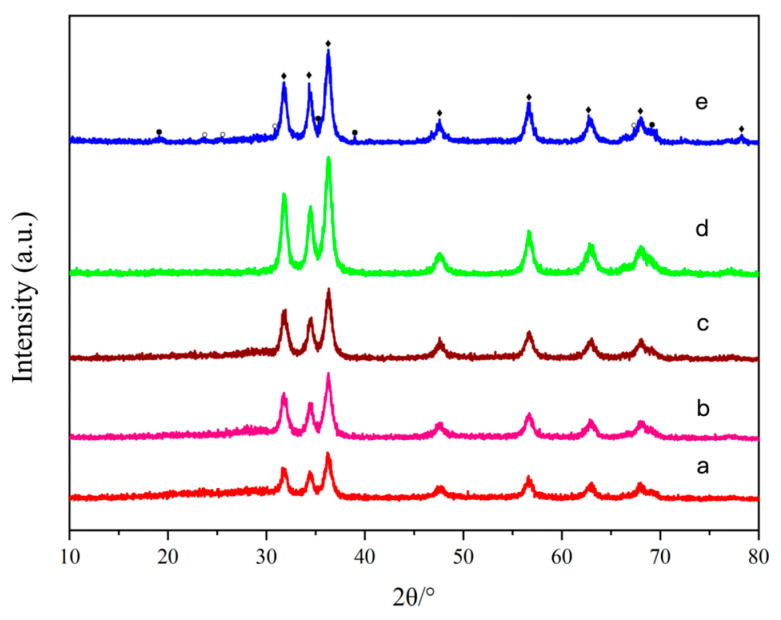
XRD patterns of nano-CuO/ZnO (TZ2) and nano-ZnO before and after desulfurization. (a) after reaction (O_2_ = 1.23%); (b) after reaction (O_2_ = 0%); (c) after reaction (O_2_ = 10%); (d) before reaction; (e) nano-ZnO after reaction. The black diamond block represents nano-ZnO after reaction, and other symbols represent unknown diffraction peaks of the unknown zinc sulfide.

As shown in Figure 7, no distinct diffraction peaks corresponding to crystalline ZnS or CuS are observed, indicating that the desulfurization products are predominantly amorphous or poorly crystallized sulfide phases.

For oxygen contents of 0% and 1.23%, the diffraction patterns after reaction exhibit an increase in background intensity in the 2θ range of 19.24–30.63°, particularly between 24.9° and 30.63°, compared with the fresh sample. This broad background feature is characteristic of amorphous or disordered phases.

Therefore, it can be reasonably inferred that the desulfurization products mainly consist of disordered sulfur-containing species, including amorphous zinc sulfides, copper sulfides, and polysulfide species. According to standard powder diffraction data (ZnO: PDF#36-1451; CuO: PDF#48-1548; ZnS: PDF#05-0566; Cu_2_S: PDF#33-0490; Cu_2_−xS: PDF#23-0958; CuS_2_: PDF#65-3322; α-S: PDF#08-0247), the corresponding crystalline peaks are absent, further confirming the amorphous nature of these sulfur-containing products.

When the oxygen content increases to 10%, the background elevation becomes more pronounced and extends to a wider range (2θ = 14.13–30.6°), indicating an increase in both the quantity and diversity of sulfur-containing species. These species likely include copper and zinc polysulfides formed during the reaction process.

It has been reported that copper sulfides can further react with elemental sulfur (S^0^) to form polysulfide chains. With increasing chain length, S–S bonds may undergo cleavage, leading to the formation of elemental sulfur (Sn), particularly under oxidative conditions [16].

From a mechanistic perspective, H_2_S initially reacts with the catalyst to form metal sulfides. In the presence of oxygen, H_2_S is partially oxidized to elemental sulfur, which subsequently reacts with metal sulfides to form polysulfides. Meanwhile, oxygen promotes the cleavage of polysulfide chains, resulting in the regeneration of elemental sulfur.

**Figure 8:**
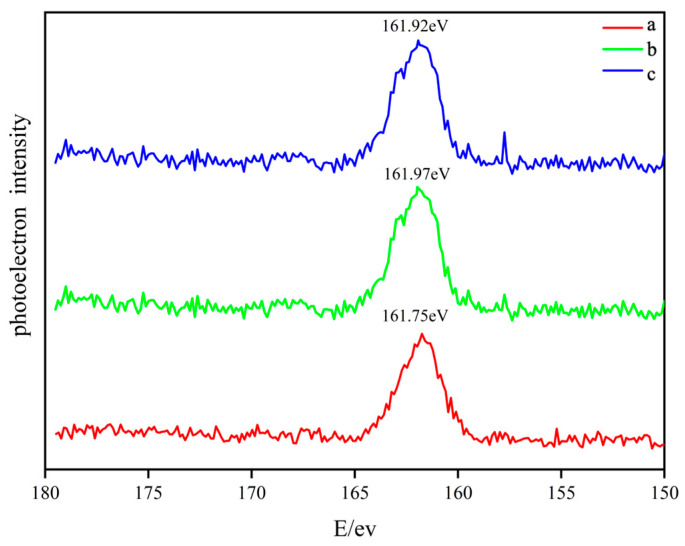
S 2p XPS spectra of nano-CuO/ZnO (TZ2) after desulfurization under different oxygen concentrations: (a) 0% O_2_; (b) 1.23% O_2_; (c) 10% O_2_.

Figure 8 (a–c) presents the S 2p XPS spectra of nano-CuO/ZnO (TZ2) after desulfurization under oxygen partial pressures of 0%, 1.23%, and 10%, respectively. As shown in Figure 8, the S 2p peaks are located at binding energies of 161.75 eV (0% O_2_), 161.97 eV (1.23% O_2_), and 161.92 eV (10% O_2_). In all cases, the S 2p spectra exhibit asymmetric peak profiles with a pronounced contribution on the higher binding energy side, indicating the coexistence of sulfur species with multiple oxidation states in the desulfurization products [16].

Notably, the relative intensity of sulfur species at higher binding energies is significantly enhanced under all conditions, suggesting the formation of oxidized sulfur species, particularly polysulfides. Furthermore, in Figure 8 (b) (1.23% O_2_), two additional shoulder peaks appear at 162.82 eV and 163.78 eV. These peaks become more prominent in Figure 8c (10% O_2_), indicating that increasing oxygen content further promotes the formation of high-oxidation-state sulfur species. These species are likely associated with polysulfide chains or partially oxidized sulfur species. The presence of oxygen facilitates the generation of polysulfides and possibly elemental sulfur.

The XRD patterns of nano-CuO/ZnO (TZ2) after desulfurization under different oxygen conditions are shown in Figure 7 (a–c). However, no distinct diffraction peaks corresponding to crystalline elemental sulfur are observed in the XRD patterns of nano-CuO/ZnO (TZ2) after desulfurization under different oxygen conditions (Figure 7 (a–c), corresponding to O_2_ = 1.23%, 0%, and 10%), suggesting that sulfur predominantly exists in an amorphous or highly dispersed state.

Combined with the XRD, XPS, and TG analyses of the fresh samples, it can be confirmed that Cu doping reduces the particle size of the desulfurizer and decreases the concentration of oxygen vacancies on the surface. The reduction in oxygen vacancies is evidenced by the increased proportion of lattice oxygen and the decreased Zn:lattice O ratio obtained from XPS analysis, as well as the diminished chemisorbed oxygen species observed in TG profiles. Since oxygen vacancies are generally considered as active sites for oxygen adsorption and activation, their decrease is expected to limit the formation of reactive oxygen species under oxygen-containing conditions. Therefore, the oxidation capability toward H_2_S may be relatively weakened.

### 2.3. Effect of Cu Doping on the Desulfurization Mechanism

#### 2.3.1. MS–H_2_S–TPD (TPSR) Analysis

**Figure 9:**
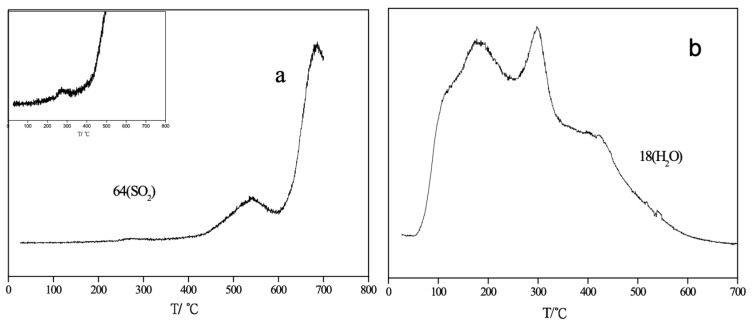
MS–H_2_S–TPD profiles of nano-CuO/ZnO (TZ2): (**a**) *m*/*z* = 64 (SO_2_); (**b**) *m*/*z* = 18 (H_2_O).

The mass spectrometry profiles of nano-CuO/ZnO (TZ2) obtained from MS–H_2_S–TPD analysis are shown in Figure 9. The desorption signal with a mass-to-charge ratio (*m*/*z*) of 64 is assigned to SO_2_ (Figure 9a). As illustrated in Figure 9a, three distinct SO_2_ desorption peaks appear at different temperature ranges, indicating the presence of sulfur species with three different chemical environments in the desulfurization products formed at room temperature.

The high-temperature SO_2_ desorption peaks at 541 °C and 685 °C are attributed to the oxidation of sulfur species coordinated with Zn and Cu on the surface, as well as sulfur species present in bulk ZnS or different crystalline forms of ZnS. These sulfur species are oxidized by reactive oxygen species derived from lattice oxygen or from water at elevated temperatures. Compared with nano-ZnO, the introduction of Cu leads to a shift in these SO_2_ desorption peaks toward lower temperatures, which can be ascribed to the reduced particle size and increased number of surface-active sites, thereby enhancing the reactivity of sulfur species.

In addition, a third SO_2_ desorption peak is observed at approximately 270 °C, corresponding to sulfur species that are more readily oxidized than ZnS or CuS formed by the adsorption of H_2_S on surface-active sites. Considering that copper sulfides tend to form polysulfide structures and that Cu species are highly dispersed as a monolayer-like CuO on the ZnO surface, this low-temperature peak is attributed to the combined contribution of copper and zinc polysulfides. These polysulfide species exhibit higher reactivity and can be oxidized at relatively lower temperatures.

Figure 9b shows the MS–H_2_S–TPD profile with *m*/*z* = 18, corresponding to H_2_O. Three H_2_O desorption peaks are observed at 176 °C, 299 °C, and 420 °C, which can be assigned to physically adsorbed water, water generated during the desulfurization reaction, and water released from the desorption of surface hydroxyl groups, respectively.

#### 2.3.2. Mechanism of H_2_S Removal over Nano-CuO/ZnO

Under room-temperature and oxygen-free conditions, the removal of H_2_S over Cu-doped nano-CuO/ZnO involves not only physical adsorption but is predominantly governed by redox-active chemisorption on the catalyst surface. The incorporation of Cu significantly reduces the particle size of the desulfurizer, increases the specific surface area, and enhances the number of surface-active sites. In particular, Cu species exhibit a strong affinity toward sulfur, which promotes the adsorption and activation of H_2_S, thereby improving the desulfurization performance at room temperature.

Although Cu doping leads to a decrease in oxygen vacancy concentration, which is generally unfavorable for oxidation reactions, the desulfurization performance is still enhanced. This indicates that the reaction mechanism is not dominated by oxygen vacancy-mediated oxidation, but rather by the intrinsic reactivity of Cu species toward sulfur-containing molecules. Due to its electronic configuration (3d^10^4s^1^) and multiple accessible oxidation states (Cu^0^, Cu^+^, and Cu^2+^), Cu can readily interact with H_2_S to form various copper sulfides, such as Cu_2_S, Cu_2_−xS, and CuS_2_.

More importantly, the unique reactivity of copper sulfides facilitates the formation of polysulfide intermediates. The synergistic interaction between highly dispersed Cu species and the ZnO matrix promotes the redox transformation of H_2_S into polysulfide species through a chemisorption-driven pathway. This process is fundamentally different from the conventional mechanism over pure nano-ZnO, where desulfurization is primarily governed by the formation of ZnS via oxygen vacancy-assisted adsorption and reaction.

In the CuO/ZnO system, the reaction pathway shifts toward a polysulfide-mediated mechanism, in which H_2_S is first adsorbed and activated on Cu–ZnO surface sites, followed by the formation of intermediate sulfide species and subsequent transformation into polysulfides. These polysulfide species exhibit higher reactivity and play a key role in the overall desulfurization process. Therefore, the enhanced performance of nano-CuO/ZnO can be attributed to the combined effects of increased surface-active sites, strong Cu-S interactions, and the formation of highly reactive polysulfide intermediates [13,14,20].

Mechanistic analysis based on combined experimental evidence indicates that the desulfurization pathway over nano-CuO/ZnO is fundamentally different from that of pure nano-ZnO.

First, MS–H_2_S–TPD results (Figure 9) reveal an additional low-temperature SO_2_ desorption peak (~270 °C), which cannot be attributed to conventional ZnS or CuS species [16,19]. This peak is assigned to highly reactive sulfur species, suggesting the formation of polysulfide intermediates. This conclusion is further supported by the S 2p XPS spectra (Figure 8), which exhibit multiple sulfur species with different oxidation states, indicating the coexistence of complex sulfur compounds rather than simple sulfides.

Second, XPS and TG analyses demonstrate that Cu doping significantly reduces the concentration of oxygen vacancies in the ZnO matrix. However, despite this decrease, the desulfurization performance is markedly improved. This observation contradicts the conventional oxygen vacancy-mediated oxidation mechanism, indicating that oxygen vacancies are not the dominant active sites in the CuO/ZnO system.

Third, Cu species, owing to their multiple oxidation states (Cu^0^/Cu^+^/Cu^2+^) and strong affinity toward sulfur, can directly participate in redox reactions with H_2_S to form various copper sulfides. These sulfides can further transform into polysulfide intermediates, which are known to exhibit higher reactivity.

In addition, Cu doping leads to reduced particle size and increased surface-active sites, enhancing the adsorption and activation of H_2_S. The synergistic effect between highly dispersed Cu species and the ZnO matrix promotes redox-driven chemisorption reactions on the catalyst surface.

Therefore, it can be concluded that, under oxygen-free conditions, H_2_S removal over nano-CuO/ZnO is predominantly governed by a redox-active chemisorption mechanism involving the formation of polysulfide intermediates, rather than by oxygen vacancy-mediated oxidation.

## 3. Experiment

### 3.1. Chemicals and Instruments

The chemical reagents used in this study were of analytical grade and used without further purification. The specific reagents and their sources are listed as follows: Zinc nitrate hexahydrate (Zn(NO_3_)_2_·6H_2_O), used for the synthesis of nano-ZnO, was purchased from Sigma-Aldrich (St. Louis, MO, USA). Urea (CO(NH_2_)_2_), used as the precipitating agent, was also obtained from Sigma-Aldrich (St. Louis, MO, USA). Hydrogen sulfide (H_2_S), supplied by a standard gas generator (Beijing Beifen-Ruili Analytical Instrument Co., Ltd., Beijing, China), was used as the reaction gas to evaluate desulfurization performance. Oxygen (O_2_), provided by a high-purity gas supplier (Air Liquide, Paris, France), was used for the oxidation reaction. Other gases and reagents, including high-purity nitrogen (N_2_), hydrogen chloride (HCl), and sodium chloride (NaCl), were obtained from Sinopharm Chemical Reagent Co., Ltd. (Shanghai, China).

The main instruments used in this study are as follows: An X-ray diffractometer (XRD, D/MAX-2500), manufactured by Rigaku Corporation (Tokyo, Japan), was used to characterize the crystal structure of nano-ZnO. A transmission electron microscope (TEM, JEM-2100), supplied by JEOL Ltd. (Tokyo, Japan), was employed to observe the morphology and particle size of the samples. X-ray photoelectron spectroscopy (XPS, PHI 5000), produced by Physical Electronics (Chanhassen, MN, USA), was used to analyze the surface chemical states of the samples. A thermogravimetric analyzer (TGA, DTG-60), manufactured by Shimadzu Corporation (Kyoto, Japan), was used to evaluate the thermal stability of the desulfurizer at different temperatures. A gas chromatograph (GC, 7890A), supplied by Agilent Technologies (Santa Clara, CA, USA) and equipped with a thermal conductivity detector (TCD), was used to analyze the concentration changes of H_2_S in the reaction gas. A mass spectrometry system (MS-H_2_STPSR, Autochem 2910), manufactured by Micromeritics (Norcross, GA, USA), coupled with an Omnistar mass spectrometer (Balzers QMG, Balzers Instruments, Balzers, Liechtenstein), was used for mass spectrometric analysis to identify intermediate products during the desulfurization process.

### 3.2. Preparation of Nano-CuO/ZnO Desulfurizer

Nano-CuO/ZnO desulfurizers were synthesized via a homogeneous precipitation method based on our previous work on the preparation of nano-ZnO and nano-CuO using nitrate precursors and urea.

Zn(NO_3_)_2_·6H_2_O, Cu(NO_3_)_2_·3H_2_O, and urea (CO(NH_2_)_2_) were separately dissolved in deionized water and filtered. The Zn(NO_3_)_2_ and Cu(NO_3_)_2_ solutions were then mixed according to the desired stoichiometric ratios. A total of 17.5 g of urea was added as the precipitating agent, maintaining a molar ratio of urea to total metal ions of 3.5:1.

The molar ratios of Cu^2+^ to Zn^2+^ in samples TZ1, TZ2, TZ3, and TZ4 were controlled at 1:50, 1:18.40, 1:11.06, and 1:6.58, respectively.

The mixed solution was transferred into a high-pressure reactor and heated at 110 °C for 1 h. After the reaction, the precursor was filtered and washed with deionized water until no NO_3_^−^ was detected in the filtrate (confirmed by the brown ring test). The precursor was then dried at 120 °C for 2 h and calcined at 260 °C for 1 h to obtain the nano-CuO/ZnO desulfurizer.

### 3.3. Evaluation of Desulfurization Performance

The desulfurization performance of nano-CuO/ZnO was evaluated using a self-built fixed-bed continuous-flow reactor. The as-prepared nano-CuO/ZnO powders were first pressed into pellets under a pressure of 20 MPa, then crushed and sieved to obtain particles with a size range of 250–425 μm. A fixed amount (0.5 g) of the sample was loaded into the center of a quartz tubular reactor (450 mm in length and 10 mm in inner diameter), ensuring that it was located within the isothermal zone.

Under a flow of high-purity nitrogen, the system was operated at atmospheric pressure and room temperature with a fixed gas hourly space velocity. A simulated gas mixture (N_2_ + H_2_S) was introduced into the reactor, with the H_2_S concentration controlled at 1852 ± 0.1 mg·m^−3^. Gas samples at both the inlet and outlet were analyzed every 10 min using gas chromatography.

**Figure 10:**
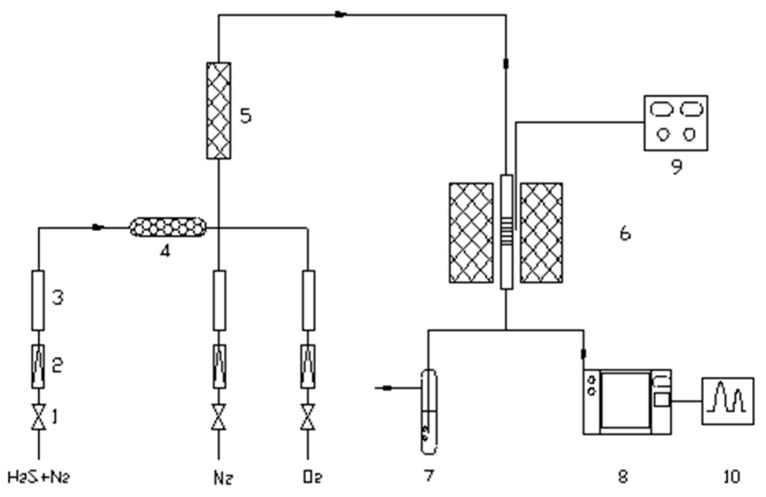
Schematic diagram of the desulfurization reaction system: (1) pressure-reducing valve; (2) flow stabilizer; (3) flow meter; (4) desiccant; (5) gas mixing chamber; (6) tubular furnace; (7) tail gas absorption bottle; (8) gas chromatograph; (9) temperature controller; (10) data acquisition system.

The breakthrough time was defined as the duration from the introduction of the reaction gas until the outlet H_2_S concentration reached 1.00 mg·m^−3^. When the outlet concentration reached this threshold, the desulfurization reaction was terminated, and the spent desulfurizer was collected for further characterization (Figure 10).

## 4. Conclusions

In this study, nano-CuO/ZnO desulfurizers were successfully synthesized via a homogeneous precipitation method, and the effects of Cu doping on their microstructure, oxygen species, desulfurization performance, and reaction mechanism were systematically investigated.

The results reveal several important new findings. First, Cu doping plays a unique structural regulation role by simultaneously reducing particle size, decreasing oxygen vacancy concentration, and increasing the specific surface area of nano-ZnO. This behavior differs from conventional strategies that rely solely on increasing oxygen vacancies to enhance activity.

Second, at room-temperature and oxygen-free conditions, the introduction of a small amount of Cu significantly enhances the desulfurization performance, with the H_2_S removal capacity nearly doubling compared with pure nano-ZnO. This improvement is attributed to the synergistic interaction between highly dispersed Cu species and the ZnO matrix, rather than a simple additive effect.

Third, a distinct desulfurization mechanism is identified. Unlike pure nano-ZnO, where adsorption is mainly governed by oxygen vacancy-assisted processes, the CuO/ZnO system predominantly follows a redox-driven chemisorption pathway. In this mechanism, Cu species facilitate the formation of highly reactive polysulfide intermediates, which play a key role in enhancing desulfurization efficiency.

Overall, this work not only demonstrates the effectiveness of Cu doping in improving room-temperature desulfurization performance but also provides new insights into the structure–property–mechanism relationship of ZnO-based desulfurizers, offering guidance for the rational design of advanced desulfurization materials.

## Figures and Tables

**Table 1:** Molar ratios of Cu to Zn in nano-CuO/ZnO desulfurizers.

Sample	Concentration of Cu^2+^ and Zn^2+^ (mg·L^−1^)		Cu: Zn Molar Ratios
	Cu^2+^	Zn^2+^	
TZ1	31.75	1575.5	1:50
TZ2	81.70	1518.4	1:18.40
TZ3	87.60	978.2	1:11.06
TZ4	294.5	1956	1:6.58

**Table 2:** Specific surface area and pore structure parameters of nano-CuO/ZnO desulfurizers.

Sample	Specific Surface Area (m^2^·g^−1^)	Pore Volume (cm^3^·g^−1^)	Average Pore Diameter (nm)
TZ1	87.86	0.4202	19.13
TZ2	102.34	0.4735	18.51
TZ3	86.56	0.3902	18.03
TZ4	72.36	0.3678	20.33
Nano-ZnO	46.86	0.3447	16.30

**Table 3:** XPS data of surface elements for ZnO and nano-CuO/ZnO.

Sample	ZnO (14.3 nm)	Nano-CuO/ZnO
Zn 2p binding energy (eV)	1021.78	1021.74
Cu 2p_3_/_2_ binding energy (eV)	—	933.39
O 1s binding energies (eV)	530.161, 531.593	530.160, 531.880
Relative content of two oxygen species (%)	58.06, 41.94	74.88, 25.12
Zn:lattice O atomic ratio	1.35	1.008

## Data Availability

The data that support the findings of this study are available from the corresponding authors upon reasonable request.

## References

[1] De Angelis A. (2012). Natural gas removal of hydrogen sulphide and mercaptans. Appl. Catal. B Environ..

[2] Ryckebosch E., Drouillon M., Vervaeren H. (2011). Techniques for Transformation of Biogas to Biomethane. Biomass Bioenergy.

[3] Bandosz J.T. (2002). On the Adsorption/Oxidation of Hydrogen Sulfide on Activated Carbons at Ambient Temperatures. J. Colloid Interface Sci..

[4] Slimane R.B., Abbasian J. (2000). Regenerable Mixed Metal Oxide Sorbents for Coal Gas Desulfurization at moderate temperatures. Adv. Environ. Res..

[5] Xu H., Guo X., Seaman L.A., Harrison A.J., Obrey S.J., Page K. (2019). Thermal desulfurization of pyrite: An in situ high-T neutron diffraction and DTA–TGA study. J. Mater. Res..

[6] Deng Y., Zhang M., Mi J., Wang J., Wu M. (2025). Insights into sulfur-release behavior during high-temperature desulfurization of wet coal gas via reaction-decoupling analysis and COS-formation inhibiting mechanism for doped metals. J. Environ. Chem. Eng..

[7] Im J.W., Ahn J.H., Kim J.H., Kim D.W., Oh K.J. (2001). Study on simultaneous removal of H_2_S/NH_3_ using a three-phase fluidized bed biofilm reactor. J. Korean Soc. Environ. Eng..

[8] Button C.A., Mantheakis E., Wang G., Reaney I.M. (2025). Effect of pressure, temperature, and particle size on cold sintered ZnO for transparent thick films on polymer substrates. J. Am. Ceram. Soc..

[9] Tran Dat T., Rong C. (2014). Desulfurization of H_2_S using porous ZnO-based materials as sorbents. Abstracts of Papers of the American Chemical Society.

[10] Sharma Y., Anand V., Kumar A., Kumar R., Paul S., Dhiman V., Heera P. (2026). Recent advances in zinc oxide nanostructures: Synthesis methods, doping Effects, Structural properties and emerging applications. Results Chem..

[11] Shao C., Wang L., Li F., Jiang A. (2009). Effect of ethanol on crystal form and desulfurization performance of nano CuO. Funct. Mater..

[12] Shao C., Jiang A., Li F., Yan B., Zhou B. (2005). ZnO Nanoparticles: Surface Structure and Desulfurization Performance for H_2_S at Room Temperature. Chin. J. Inorg. Chem..

[13] Hao F., Zhang X. (2024). Adsorption Mechanism of Sulfur-Containing Gases on Metal-Doped ZnO Surfaces: A DFT Study. Surf. Sci..

[14] Kim D., Bae D., Kim Y.J., Lee S.J., Lee J.W., Yun Y., Park N.K., Kim M. (2021). Enhancement of desulfurization capacity with Cu-based macro-porous sorbents for hydrogen production by gasification of petroleum cokes. Appl. Sci..

[15] Vinod R., Junaid M.B. (2023). Hydrothermal Growth of ZnO Nanostructures with Dopants Co^2+^, Ni^2+^ and Cu^2+^-Structural and luminous characteristics. J. Lumin..

[16] Laperdrix E., Costentin G., Saur O., Lavalley J.C. (2000). Selective Oxidation of H_2_S over CuO/Al_2_O_3_: Identification and Role of the Sulfurated Species Formed on the Catalyst during the Reaction. J. Catal..

[17] Ma E., Tian X., Liu J., Guo A., Huang W., Zhang Q. (2025). Modulation of electronic metal-support interaction on Cu/ZnO by metal oxides for iso-butanol synthesis from syngas. Fuel.

[18] Shao C., Gao Y., Qi X., Yang X. (2025). Study on the Surface Structure of Nano-ZnO Desulfurizers and Their Performance and Mechanism in H2S Removal at Room Temperature. Catalysts.

[19] Kattel S., Ramírez P.J., Chen J.G., Rodriguez J.A., Liu P. (2017). Active sites for CO_2_ hydrogenation to methanol on Cu/ZnO catalysts. Science.

[20] Wang Z., Zhang N., Zhang N., Zhan Y., Yang L., Wu Z., Hu Q., Feng M., Huang X. (2026). Engineering Oxygen Vacancies and Curled Ultrathin Architecture in ZnO Nanosheets for Efficient U(VI) Removal Under Acidic Conditions. https://ssrn.com/abstract=6079656.

